# A Two-Stage Method for Target Searching in the Path Planning for Mobile Robots

**DOI:** 10.3390/s20236919

**Published:** 2020-12-03

**Authors:** Tao Song, Xiang Huo, Xinkai Wu

**Affiliations:** 1 School of Transportation Science and Engineering, Beihang University, Beijing 100193, China; songtaobuaa@buaa.edu.cn (T.S.); huoxiang@buaa.edu.cn (X.H.); 2 Beijing Advanced Innovation Center for Big Data and Brain Computing, Beihang University, Beijing 100193, China

**Keywords:** targets search, mobile robot, path planning, visual complete coverage, two-stage

## Abstract

The path planning for target searching in mobile robots is critical for many applications, such as warehouse inspection and caring and surveillance for elderly people in the family scene. To ensure visual complete coverage from the camera equipped in robots is one of the most challenging tasks. To tackle this issue, we propose a two-stage optimization model to efficiently obtain an approximate optimal solution. In this model, we first develop a method to determine the key locations for visual complete coverage of a two-dimensional grid map, which is constructed by drawing lessons from the method of corner detection in the image processing. Then, we design a planning problem for searching the shortest path that passes all key locations considering the frequency of target occurrence. The testing results show that the proposed algorithm can achieve the significantly shorter search path length and the shorter target search time than the current Rule-based Algorithm and Genetic Algorithm (GA) in various simulation cases. Furthermore, the results show that the improved optimization algorithm with the priori known frequency of occurrence of the target can further improve the searching with shorter searching time. We also set up a test in a real environment to verify the feasibility of our algorithm.

## 1. Introduction

In recent years, the application of intelligent mobile robots in a smart home system has attracted many attentions. In a home environment, mobile robots equipped with multiple sensors are often used to perform some searching tasks, such as searching for people, pets, or objects. Due to the complexity of the indoor environment with many obstacles, generating a search path plan for a specific target is a big challenge for robots. In particular, it becomes a difficult task to quickly generate an efficient search path plan for a mobile robot equipped with a camera. In fact, with the known map information, the search task using cameras is a vision coverage path planning (VCPP) problem. This problem can be regarded as a variant problem derived from the coverage path planning (CPP) problem. CPP essentially is used to determine a path that passes all points of an area or volume of interest while avoiding obstacles [1]. CPP has been widely used in many robotic applications, such as vacuum cleaning robots, construction waste recycling robots, autonomous underwater vehicles, creating image mosaics, demining robots, lawn mowers, automated harvesters, window cleaners, and inspection of complex structures [2,3,4,5,6].

A considerable body of research has studied the CPP problem (e.g., [1,6,7,8,9]), but relatively little research has been conducted on VCPP. Intuitively speaking, VCPP is a combination of solving a Watchman Route Problem (that is, a problem of computing the shortest route from Point A to Point B in a known area) and an Art Gallery Problem (that is, a problem of selecting the minimum number of observation points to completely observe a known area). Finding an optimal solution to this combination is obviously a daunting task. Wang et al. [10] used an approximation method to find a bounded suboptimal solution, which considers both traveling time and sensing time with respect to a pre-defined set of sensing positions. However, the problem of selecting the initial set of sensing positions remains open and the approach presents scalability issues when the cardinality of the set is increased. Some other algorithms have been proposed for the optimal solution of VCPP; but these algorithms are only effective with restricted assumptions, such as not considering occlusions in the field of view of the sensors [11], or the detection environment is relatively simple [12]. These solutions present two major drawbacks: (1) these methods are difficult to efficiently solve the problem in some complex scenarios; and (2) they do not consider the cost of moving from one search location to another. Please note, in a complicated indoor environment, the mobile robot moving cost between key search locations is critical and must be considered throughout the target search path planning process. Therefore, after determining key search locations, we will still need to solve a (Metric) Traveling Salesman Problem to find the shortest tour that connects all the search locations [13]. It is a well-known NP-hard problem, although it is possible to optimally solve very large TSP instances, with hundreds of locations [14].

This research aims to tackle the VCPP problem in a complex indoor environment for mobile robots. Our main work includes: (1) Propose an efficient method to determine the key locations for visual complete coverage of a two-dimensional grid map, which is constructed by drawing lessons from the method of corner detection in the image processing. As the field of view of robot camera can completely search the entire known area, the union of the area covered by the visual field of the robot camera at the key locations can achieve full coverage of the whole environment. (2) Design a planning problem for searching the shortest path that passes all key locations considering the frequency of target non-occurrence. The Ant Colony Optimization (ACO) algorithm will be used to solve this problem.

The rest of the article is structured as follows. We define the path planning problem of target search with a mobile robot in Section 2, followed by a two-stage optimization model in Section 3. Section 4 presents the detailed methods of our model. In Section 5, multiple simulation and experimentation results are conducted for comparisons and discussion. Finally, we conclude the article in Section 6.

## 2. Problem Description

The proposed VCPP solution is applied to a mobile robot with fundamental functions including motion control, sensor information fusion, simultaneous mapping, navigation capabilities, etc. Target searching is carried out by a camera mounted on a 360-degree controllable vision pan-tilt unit. Real-time images at current location will be processed by image process algorithm to extract target information. During the process, we also assume the environment is static and the locations of walls or other obstacles will not be changed.

**Figure 1:**
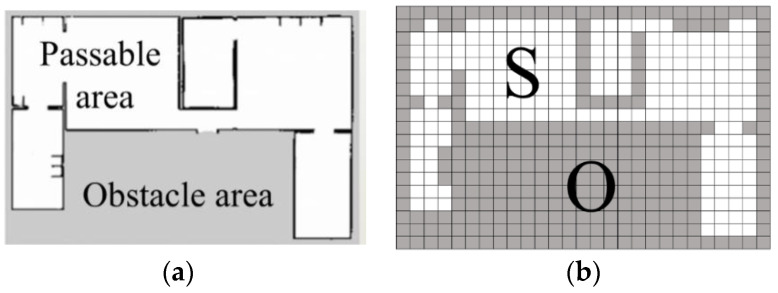
(**a**) Environment map; (**b**) grid map.

To increase the cover range of the robot camera, a 360-degree controllable vision pan-tilt unit is equipped, and the radius of search circular is assumed *r*. Therefore, the robot can detect an area with 360-degree view. Note we assume the working environment for mobile robot is known; and the grid map is derived by the widely used coverage algorithm developed by Mansouri et al. [9]. In the grid map, the side length of the square grid is determined by the size of the robot outline plus a safe distance. Therefore, the target area can be defined as a Cartesian grid with a set of all identical grids, *A*. Within *A*, the subset *O* covers all grids containing obstacles which stop the detection of the robot camera and the movement of the robot; and the subset *S* includes *N* grids which do not contain obstacles, i.e., S={1,⋯,N}, as shown in Figure 1. We assume that every passable grid is reachable for the robot.

To complete the target searching, we need to first determine an optimal path which can be passed by the robot. We then define the optimal path as a set, *P*, an ordered column vector with the first cell as the starting location of the mobile robot. As *P* is defined as an optimal path, then the moving cost associated to *P* is minimal; and the visual coverage covers all passable grids through a series of observation locations of *P*. These observation locations in *P* are defined as {$p_{1},\cdots ,p_{i},\cdots ,p_{n}$}, i.e., $P=\{p_{1},\cdots ,p_{i},\cdots ,p_{n}\}$. In addition, $p_{i}\to p_{i+1}$ is the subpath of the optimal path *P*.

**Figure 2:**
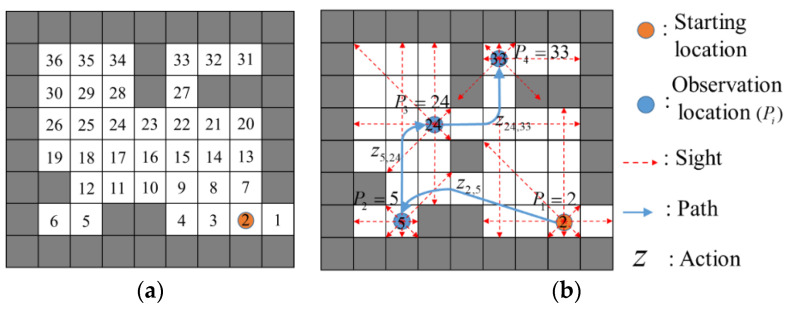
(**a**) Test map; (**b**) optimal path *P* (2→5→24→33).

An example of these steps is detailed in Figure 2. The simple test map is shown in Figure 2a, where obstructed grids are represented in black and passable ones in white. The passable grids have been marked with number 1, …, 36. As shown in Figure 2b, the optimal path *P* (2→5→24→33) is determined by a minimum length shortest path, i.e., *P* = {2,5,24,33}.

## 3. A Two-Stage Model for VCPP

Before we present the model, the list of the notations is summarized in Table 1.

### 3.1. Mathematical Model

We aim to find the optimal observation location configuration *P* with minimum moving cost by solving the following problem:

Minimize

(1) $$\sum_{i=1}^{n-1}c(z_{p_{i},p_{i+1}})$$

subject to

(2) ∪v(Z)=S

(3) $$p_{1}=g$$

(4) P⊆S

where Equation (1) is the objective function of the model. The moving cost includes the path distance generated by the subpath of *P* performed by the mobile robot. $c(z_{p_{i},p_{i+1}})$ is the moving cost associated with the subpath $z_{p_{i},p_{i+1}}$ ($p_{i}\to p_{i+1}$, $p_{i}$,$p_{i+1}\in P$).

∪v(Z) is the union of a series of visible grid sets detected by *Z*. *S* is the set of passable grids. All grids detected by the observation locations of *P* include all passable grids, as shown in Equation (2).

*g* is the starting location of the mobile robot. The first cell of *P* $p_{1}$ is set as the starting location of the mobile robot, as expressed in Equation (3).

The ordered column vector $P=\{p_{1},\cdots ,p_{n}\}$, which expresses the optimal path passed by the robot, is the decision variable. A series of grids ∈S make up the ordered column vector *P*. *P* is a subcollection of *S*, as shown in Equation (4).

### 3.2. A Two-Stage Model Design

**Figure 3:**
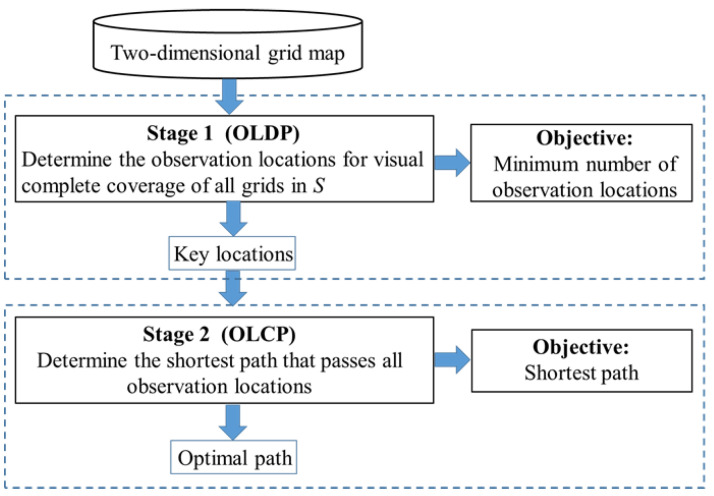
The diagram of the two-stage optimization program.

It is difficult to find an optimal subcollection *P* of *S* to address the problem of target search in a complex grid map due to the computational complexity. To improve computational efficiency, we can obtain an approximate optimal solution by structuring a two-stage optimization model. In the first stage, we focus on minimizing observation locations to achieve vision complete coverage of all grids in *S*; and in Stage II, we address the path planning problem by connecting these observation locations. The diagram of the two-stage optimization program is shown in Figure 3. In brief, in this proposed two-stage optimization program, Stage I is modeled as an observation location determination problem (OLDP); and Stage II is designed as an observation location connectivity problem (OLCP).

(1) Stage I: An OLDP model: The basic idea is to lock some observation locations by thoroughly observing all grids in *S*. The excellent method used by some authors to find a pre-defined set of sensing locations to completely observe a known area (e.g., [10,13]). However, these methods are poorly implementable when the cardinality of the set of sensing positions is large. We focus on minimizing observation locations to achieve vision complete coverage of all grids in *S*. The observation locations are defined as “key locations”. The detailed OLDP modeling is described as follows:

Minimize

(5) $$\sum_{k\in S}\alpha_{k}$$

subject to

(6) $$\alpha_{k}=\left\{ \begin{array}{l} 1\;\mathrm{if}\;k\in \Omega \\ 0\;\mathrm{otherwise} \end{array}\right.\;k\in S$$

(7) ∪v(Ω)=S Ω⊆S

(8) $$\alpha_{g}=1$$

Objective function: Equation (5) is the objective function of the OLDP model. As few observation locations as possible are obtained to solve VCPP.

Constrains: After we construct the set of key locations as Ω, we introduce a binary variable $\alpha_{k}$, where $\alpha_{k}=1$ when the grid *k* is set as a key location and $\alpha_{k}=0$ otherwise, as shown in Equation (6).

In Equation (7), ∪v(Ω) denotes the union of all visible grid sets detected by the robot passing all key locations of Ω. Equation (7) explains that the area seen by the robot passing all key locations will cover all grids in *S*.

Equation (8) explains the starting location g of the mobile robot belongs to Ω.

A heuristic method is used to solve OLDP, as will be introduced in Section 4.1.

(2) Stage II: An OLCP model: After the set of key locations Ω is obtained, we then search the shortest path which connects these key locations, which is modeled as an OLCP problem.

OLCP is similar to the traveling salesman problem (TSP). However, the classic TSP requires that the traveler departs from the starting point and returns to the starting point after passing through all locations. In our model, we search for a shortest path which visits each key location in Ω but without going back to the starting location. Therefore, our planned path will not form a closed loop, i.e., Hamiltonian cycle. To still apply the TSP model, we add a virtual location to Ω, set the distance from this virtual point to the starting location is 0 and set the distance from this virtual point to other key locations is the maximum distance between key locations. After adding a virtual location, we solve the TSP problem to plan a looped path and then remove the virtual points to get the optimized path.

In addition, a critical point for our research is that the shortest path that passes all key locations is determined considering the frequency of target non-occurrence of each location. The more difficult it is to observe the target in some locations, the less it should be prioritized to go to these locations for robot when designing the path. In other words, the higher the moving cost to reach these locations should be.

To formulate the above-mentioned frequency problem, we introduce a parameter $f_{k}$, which indicates the frequency of the robot which is unable to search for the target at the key location *k*. When the robot moves to location *k* without finding the target, the path distance to location *k* is the real moving cost for our model. The moving cost from any key location *j* to key locations *k* is estimated as the product of d(j,k) and $f_{k}$. d(j,k) is the path distance between key locations *j* and *k*.

Then, this OLCP model with frequency can be described in the following:

Minimize

(9) $$\sum_{j\in \Omega }\sum_{k\in \Omega }\left(x_{j,k}\cdot d(j,k)\cdot f_{k}\right)\;j\neq k$$

subject to

(10) $$x_{j,k}=\left\{ \begin{array}{l} 1\;j\;\mathrm{and}\;k\;\mathrm{are}\;\mathrm{connected}\\ 0\;\mathrm{oterwise} \end{array}\right.\;j,k\in \Omega ,\;j\neq k$$

(11) $$f_{k}=\frac{\frac{w_{k}}{W}}{\sum_{k\in \Omega }\left(\frac{w_{k}}{W}\right)}$$

(12) $$\sum_{j\in \Omega }x_{j,k}=1\;j,k\in \Omega ,\;j\neq k$$

(13) $$\sum_{k\in \Omega }x_{j,k}=1\;j,k\in \Omega ,\;j\neq k$$

(14) $$\sum_{j\in \Omega }\sum_{k\in \Omega }x_{j,k}\leq |\Phi |-1\;j,k\in \Phi ,\;\forall \Phi \subset \Omega ,\;2\leq |\Phi |\leq N_{\Omega }$$

In detail, the OLCP model consists of:

Objective function: Equation (9) is the objective function of the OLCR problem. It searches the optimal path with shortest distance which connects all key locations in Ω. The binary factor $x_{j,k}$ indicates whether the mobile robot moves from key location *j* to *k*. d(j,k) illustrates the distance between key locations *j* and *k*. $f_{k}$ illustrates the frequency of the robot which is unable to search for the target at the key location *k*.

Constrains: $x_{j,k}$ indicates whether the mobile robot moves from key location *j* to *k* is the decision variable in this model, as formulated by Equation (10).

$w_{k}$ is the number of test times of the robot unable to search for the target at key location *k*. W is the total number of target search test times The frequency that the robot unable to search for the target at the key location *k* is $\frac{w_{k}}{W}$. It means that the mobile robot passes the key location *k* $w_{k}$ times but cannot find the target in total *W* times tests. After normalization, this frequency value $f_{k}$ is $\frac{\frac{w_{k}}{W}}{\sum_{k\in \Omega }\left(\frac{w_{k}}{W}\right)}$, as shown in Equation (11). In the absence of multiple test data for searching the target in the working environment (i.e., no prior knowledge of target non-appearance frequency), $f_{k}$ is set to 1.

Equations (12) and (13) explain that for each key location, only one path enters the key location and only one path moves out of the key location. Equation (14) guarantees that the model does not have any sub-circuit solutions. In Equation (14), Φ is a subset of set Ω. The number of key locations in Φ is within 2 and $N_{\Omega }$.

The Ant Colony Optimization algorithm (ACO) is used to solve OLCP, as will be introduced in Section 4.2.

## 4. Solving Methods

### 4.1. Stage I: A Heuristic Method

**Figure 4:**
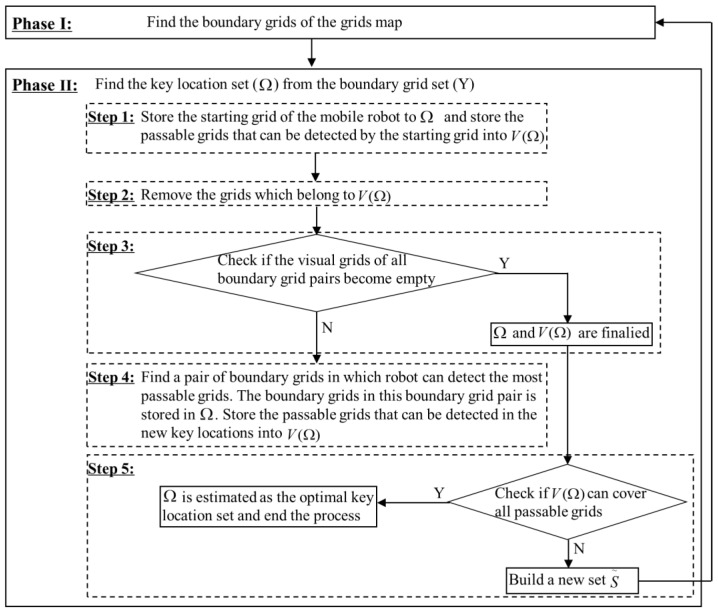
The flow chart of the method to pick out key locations in the grid map.

It is challenging to find the key locations in a complex grid map. Through our observations, we find that searching key locations using the boundary grids will significantly reduce the complexity of the algorithm. Therefore, we propose the following two-phase heuristic method: first, we use the pixel-level corner detection method in the image processing to find the boundary grids in the “relative macro” grid map scene used by the mobile robot; and second, we search the key locations in these boundary grids, instead of a complex analytical solution process. The detailed procedure is described in the following steps and the flowchart in Figure 4.

Phase I: Apply the pixel-level corner detection method to find the boundary grid set (Y) of the grids map.

(15) $$\left\{ \begin{array}{l} i\in Y\;E(i)>0\\ i\notin Y\;E(i)=0 \end{array}\right.\;i\in A$$

where:

(16) $$E(i)=e(x_{i},y_{i})\cdot [\sum_{u}\sum_{v}{\left(e(x_{i}+u,y_{i}+v)-e(x_{i},y_{i})\right)}^{2}]\;i\in S,u\in \{-1,0,1\},v\in \{-1,0,1\}$$

(17) $$e(x_{i},y_{i})=\left\{ \begin{array}{l} 0\;i\in O\\ 1\;i\in S \end{array}\right.$$

**Figure 5:**
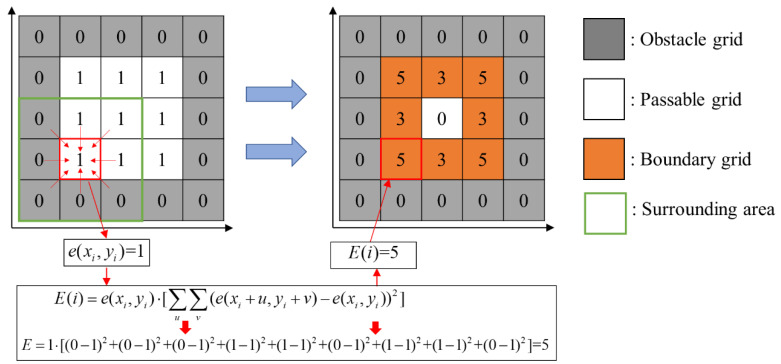
The calculation process example of the boundary judgment parameter for each grid.

As presented in Equation (15), if the boundary judgment parameter for grid *i*, i.e., E(i) calculated by Equation (16), is greater than 0, the grid *i* is the boundary grid, i.e., i∈Y. *A* is the set of all identical grids of the gird map. In Equation (16), $x_{i}$ and $y_{i}$ are the coordinates of grid *i*; and $e(x_{i},y_{i})$ is the attribute value of grid *i*. The attribute value of obstacle grid (set *O*) is 0, and the attribute value of passable grid (set *S*) is 1, as formulated in Equation (17). $e(x_{i}+u,y_{i}+v)$ is the attribute value of surrounding grid of the grid *i*. u and v are the offsets of the abscissa and ordinate respectively, which belong to {−1,0,1}. Figure 5 presents a simple example.

Phase II: Find the key location set (Ω) from the boundary grid set (Y). As there are multiple boundary grids, the computational complexity of searching the key locations from all boundary grids is still high. The basic idea is to first form boundary grids into boundary pairs ({i,j}, i,j∈Y), and passable grids which can be detected by the boundary grids *i* and *j* are defined as the visible grids of boundary grid pair $v_{\left\{i,j\right\}}$, where i,j∈Y. Then, the pairs of boundary grids in which the robot can detect the most passable grids are stored into the key location set Ω. The detailed procedure is described in the following:

- Step 1: store the starting grid of the mobile robot to the key location set Ω; and store the passable grids that can be detected by the starting grid into the visible grid set of the key locations, i.e., V(Ω).
- Step 2: remove the grids which belong to V(Ω) in the visual grids of all boundary grid pairs.
- Step 3: check if the visual grids of all boundary grid pairs become empty. If so, the key location set (Ω) and visible grid set V(Ω) are finalized, and go to Step 5. Otherwise, go to Step 4.
- Step 4: find a pair of boundary grids in which robot can detect the most passable grids. If there are multiple boundary grid pairs in which robot can detect the most passable grids, we select the boundary grid pair which is closest to the starting grid. The boundary grids in this boundary grid pair is stored in the key location set (Ω). Store the passable grids that can be detected in the new key locations into the visible grid set of key locations (V(Ω)). Then, return to Step 2.
- Step 5: check if the visible grid set of key locations (V(Ω)) can cover all passable grids. If so, the key location set (Ω) is estimated as the optimal key location set and end the process. If V(Ω) cannot cover all passable grids, we need to build a new set $\tilde{S}$. The new set $\tilde{S}$ only retains these passable grids which cannot be detected in the original set *S*. Replace set *S* with set $\tilde{S}$ and add these passable grids in the original set *S* that already be detected to set *O*. Then, repeat the Phase I.

**Figure 6:**
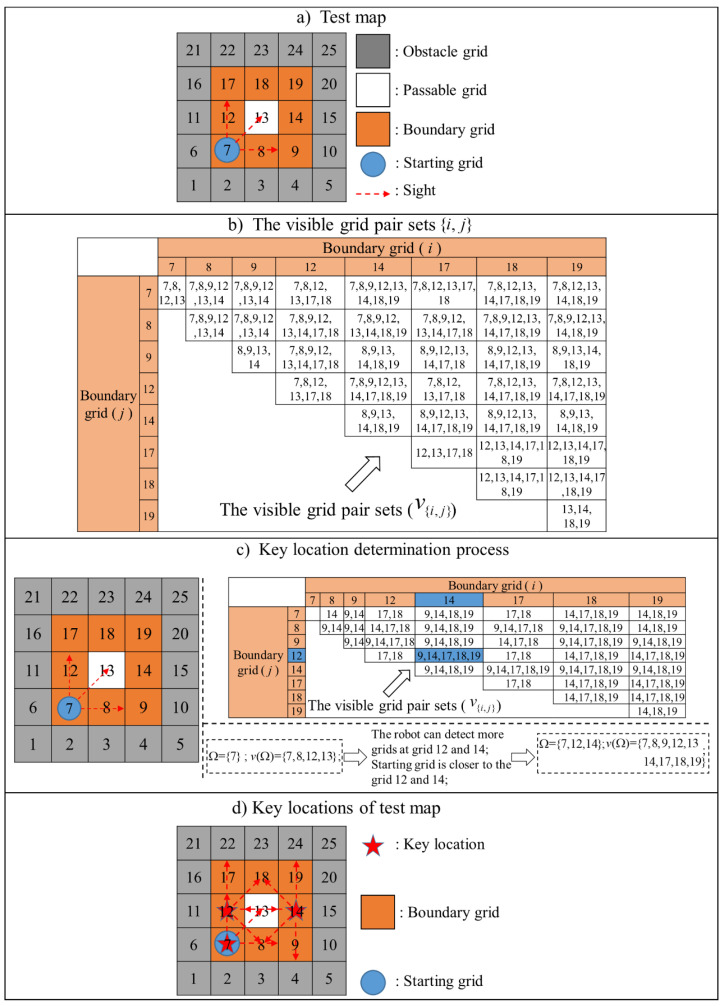
The repetitive process of key location determination.

An example of key location determination algorithm is detailed in Figure 6. Figure 6a presents a simple test map, where obstructed grids are in black. We first use the pixel-level corner detection method described in Phase I to find the boundary grids, and set the starting grid 7. According to the sign of the robot, the passable grids that can be detected by the starting grid are {7,8,12,13}. Then, boundary grids are formed into boundary pairs ({i,j}, i,j∈Y), and passable grids which can be detected by the boundary grids *i* and *j* are defined as the visible grid pair sets $v_{\left\{i,j\right\}}$, where i,j∈Y (see Figure 6b). The procedure to find the key location set (Ω) from the boundary grid set is described in Figure 6c. The detailed procedure is described in the following steps:

(1) Set the starting grid 7 to the key location set Ω; and store the passable grids that can be detected ({7,8,12,13}) into the visible grid set of the key locations V(Ω).
(2) Remove the grids belonging to V(Ω) in the visual grids of boundary grid pairs $v_{\left\{i,j\right\}}$.
(3) If the visual grids of all boundary grid pairs are not empty, then go to next step.
(4) Find some pair of boundary grids in which the robot can detect the most passable grids (i.e., {12,14}, {14,17}, {8,18}, {9,18}, {14,18}). Since {12,14} is the closest one, grids 12 and 14 are stored into the key location set (Ω). Then the new key locations set is ({9,14,17,18,19}).

Check if the visible grid set of key locations (V(Ω)) can cover all passable grids. If cation set (Ω) is estimated as the optimal key location, set (i.e., {7,12,14}), as shown in Figure 6d.

### 4.2. Stage II: Ant Colony Optimization Algorithm (ACO)

Section 4.1 essentially provides the key locations set of the grid map Ω. Given the key locations and the distance between each pair of key locations, we then need to solve the shortest path for the robot to visit each key location from the starting location.

To solve this problem, we first use the A-star algorithm to determine the distance between two key locations in the grid map [15]. More importantly, to improve efficiency, we propose to apply the ACO to find a near-optimal solution. ACO has been widely applied in many fields (e.g., [16,17,18]).

The following explains the detailed calculation steps for ACO.

- Step 1: Initialization. Initialize the number of ants *M*, pheromone importance factor κ, pheromone concentrations $\tau_{0}$, importance factor of heuristic function λ, volatile degree of pheromone ρ, the total amount of pheromone released *Q*, the maximum number of iterations $t_{\max}$, and the initial number of iterations t=1.
- Step 2: Construct the solution space. The ants are randomly placed at different starting locations. Each ant (θ(θ=1,⋯,M)) determines the next location ∈Ω to visit based on the probability $\chi_{i,j}^{\theta }(t)$ calculated by Equations (18) and (19) until all the ants have reached all locations ∈Ω.

(18) $$\chi_{i,j}^{\theta }(t)=\left\{ \begin{array}{ll} \frac{{\left[\tau_{i,j}(t)\right]}^{\kappa }\cdot {\left[\eta_{i,j}(t)\right]}^{\lambda }}{\sum_{\varepsilon \in allow_{\theta }}{\left[\tau_{i,\varepsilon }(t)\right]}^{\kappa }\cdot {\left[\eta_{i,\varepsilon }(t)\right]}^{\lambda }},\; & \varepsilon \in allow_{\theta }\\ 0, & \varepsilon \notin allow_{\theta } \end{array}\right.$$

(19) $$\eta_{i,j}(t)=\frac{1}{d(i,j)\cdot f_{j}}$$

where:

$\chi_{i,j}^{\theta }(t)$ is the probability of ant θ transferring from location *i* to *j* at iteration *t*.
$\tau_{i,j}(t)$ is the pheromone concentration on the connection path between *i* and *j* at *t*.
d(i,j) illustrates the distance of those key locations *i* and *j*.
$f_{i}$ is the frequency of the robot which is unable to search for the target at the location *j*.
${\mathrm{allow}}_{\theta }$ is the set of locations to be visited for ant θ.
κ is the pheromone importance factor. The larger κ, the more significant the role of pheromone concentration in the path selection.
λ is the critical factor of heuristic function. The larger λ, the more significant the role of the heuristic function in the path selection. That is, ants will move to key locations with short distances with a high probability.

- Step 3: Determine the optimal solution. The path length of each ant $L_{\theta }(\theta =1,\cdots ,M)$ will be calculated according to the distance between each key position, and all paths are recorded in the record table. Meanwhile, we record the optimal solution in the current number of iterations.
- Step 4: Update the pheromone. The pheromone concentration on the connection path between key locations is updated according to Equations (20)–(22).

(20) $$\tau_{i,j}(t+1)=(1-\rho )\cdot \tau_{i,j}(t)+\Delta \tau_{i,j}\;0<\rho <1$$

(21) $$\Delta \tau_{i,j}=\sum_{\theta =1}^{M}\Delta \tau_{i,j}^{\theta }$$

(22) $$\Delta \tau_{i,j}^{\theta }=\left\{ \begin{array}{l} \frac{Q}{L_{\theta }}\;\mathrm{Ant}\;\theta \;\mathrm{transfers}\;\mathrm{from}\;i\;\mathrm{to}\;j\\ 0\;\mathrm{Otherwise} \end{array}\right.$$

where: *Q* represents the total amount of pheromone released, and it is constant. $L_{\theta }$ is the distance of the ant θ traverse path.

- Step 5: Judge the termination conditions. If the maximum iteration is met, stop calculation and output the optimal result. Otherwise, iteration increases by one, the records of the ant paths are cleared and return to Step 2.

## 5. Evaluation

### 5.1. Case Scenarios

**Figure 7:**
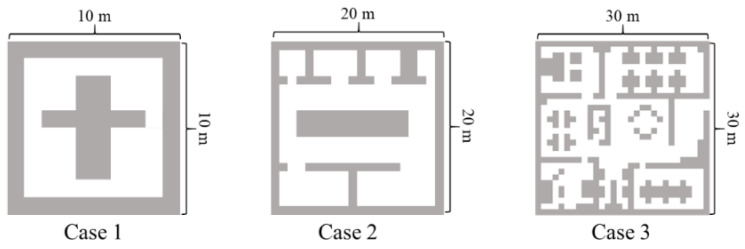
Case area.

We evaluated our algorithm on maps with grid sizes ranging from 10 × 10, 20 × 20, 30 × 30 cells (i.e., the environment is known), as shown in Figure 7. We used three different maps (cases) to verify our method. We define a grid of grid maps with a side length of 1 m and an area of 1 square meter. Therefore, these three cases are 100 square meters, 400 square meters, and 900 square meters, respectively.

The visual sensing parameter of the mobile robot (radius of circular measurement sectors) *r* is set as 15 m. The mobile robot will perform the target search process at a uniform speed (0.6 m/s) from the starting location of the map. The algorithm was run in an i7 processor @ 3.4 GHz, 32.00 GB RAM computer with a Windows 10 64bit operation system. For optimization, we used MATLAB R2015b.

**Figure 8:**
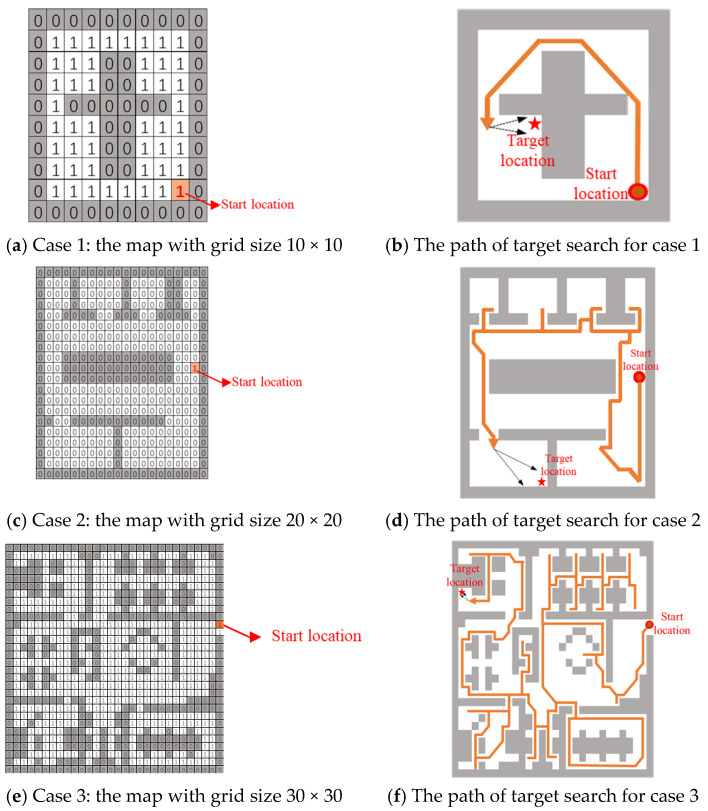
Generated path of target search for 3 cases.

Firstly, we assume no prior knowledge of target frequency, i.e., we do not have test data for searching the target in the working environment. In this simulation, a target is randomly placed in a passable grid of the grid map. Then we obtained the target search path through our optimization algorithm (OA), as shown in Figure 8. The total path length in case 1 brought by OA is 12 m. The time used in this target search in case 1 is 20 s. The total path length in case 2 brought by OA is 76 m. The time used in this target search in case 2 is 126.67 s. The total path length in case 3 brought by OA is 219 m. The time used in this target search in case 3 is 365 s.

### 5.2. Result Comparisons with Two Other Algorithms

We then compare our results with two other algorithms: Rule-based Algorithm (RBA) and Genetic Algorithm (GA) using the above three cases.

(1) Rule-based Algorithm (RBA): Rule-based Algorithm is a heuristic algorithm, including the internal spiral coverage algorithm (ISC) for full coverage path planning of sweeping robot and the frontier-based algorithm for autonomous exploration of the robot, etc. The inner spiral coverage algorithm was initially proposed by Butler et al. [19] based on the contact sensor algorithm. The basic idea of the internal spiral coverage algorithm is that the mobile robot advances clockwise or counterclockwise to traverse the entire work environment. The frontier-based algorithm for autonomous exploration of the robot was proposed by Brian et al. [20]. Frontiers are regions on the boundary between unexplored and explored space. To gain the newest and useful information, the robot must move to the borders and explore again.
(2) Genetic Algorithm (GA): GA is an intelligent algorithm that was proposed by Holland in 1975 [21]. It is noted that GA has a strong global search ability and is suitable for discrete variable optimization problems. It imitates biological inheritance and evolution and introduces the concept of survival of the fittest in evolution into the algorithm.

#### 5.2.1. Average Target Search Time

**Figure 9:**
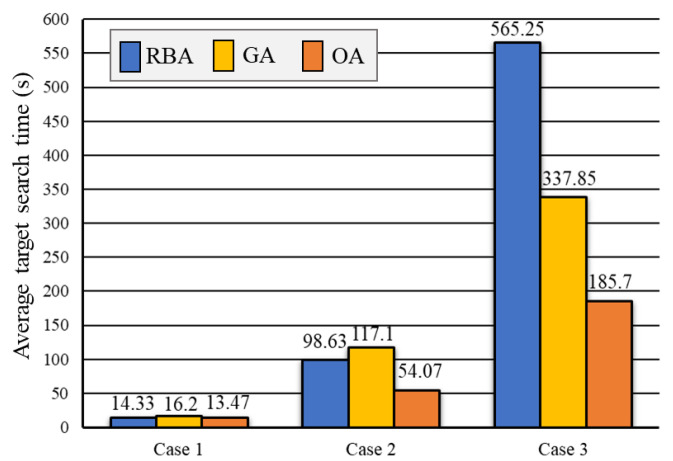
Average target search time for 3 cases obtained by Rule-based Algorithm (RBA), Genetic Algorithm (GA), and optimization algorithm (OA).

We set up 100 tests for each case. In each test, a target is randomly placed in a passable grid of the grid map. The mobile robot searches for the target at the same speed (0.6 m/s) according to the planned path obtained by RBA, GA, and OA. Then, we present the average time used in the process of target search in a total of 300 tests of 3 cases obtained by RBA, GA, and OA, as shown in Figure 9.

**Figure 10:**
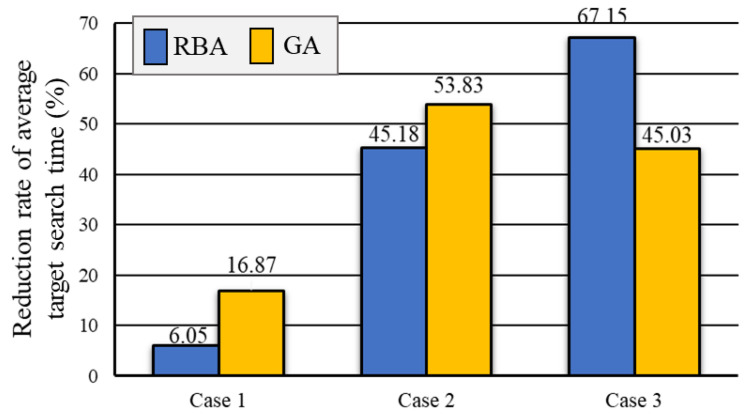
The reduction rate of average target search time by OA, RBA, and GA.

As shown in Figure 9, in Case 1, the average time obtained by RBA is 14.33 s, GA is 16.2 s, and our method OA is 13.47 s. In Case 2, the average time obtained by RBA is 98.63 s, GA is 117.1 s, and our method OA is 54.07 s. In Case 3, the average time obtained by RBA is 565.25 s, GA is 337.85 s, and OA is 185.7 s. The results show that the average time of target searching is significantly reduced with the help of our optimization algorithm. Note, the larger and more complex the environment map, the more pronounced the decline of the average time of target searching. The reduction rates of the average time of for three cases by three methods are also presented in Figure 10.

#### 5.2.2. Path Length of Vision Complete Coverage of Passable Area

**Figure 11:**
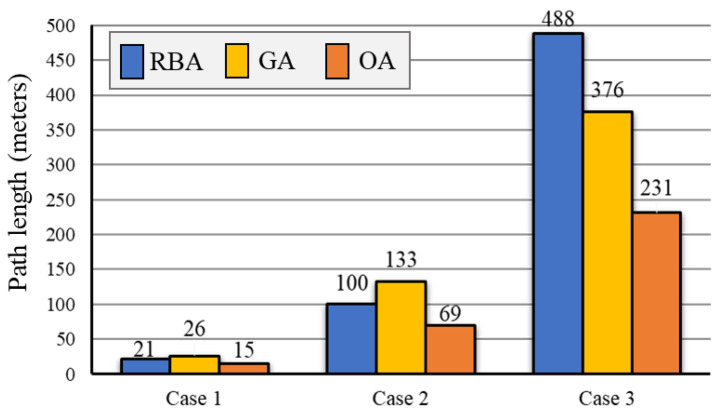
Path length of 3 cases obtained by RBA, GA, and OA.

**Figure 12:**
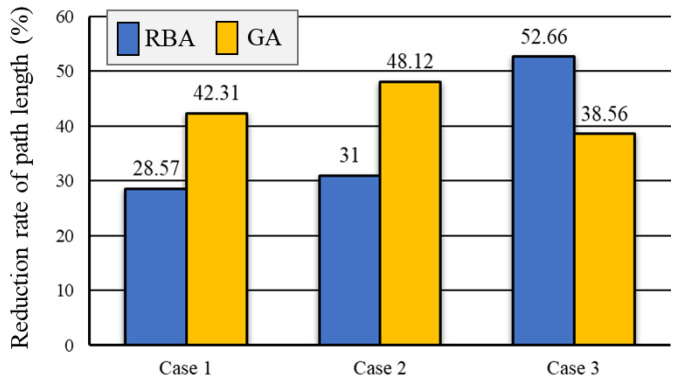
The distance reduction rate of path obtained by OA relative to RBA and GA.

We present the total path length of 3 cases obtained by RBA, GA, and OA, as shown in Figure 11. The total path length in Case 1 brought by RBA is 21 m, by GA is 26 m, and by OA is 15 m. The total path length in Case 2 obtained by RBA is 100 m, by GA is 133 m, and by OA is 69 m. The total path length in the Case 3 obtained by RBA is 488 m, by GA is 376 m, and by OA is 231 m. The path distance reduction rates are shown in Figure 12. The comparisons show that the proposed OA algorithm can effectively reduce the length of full searching path.

### 5.3. Results with the Prior Knowledge of Target Frequency

**Figure 13:**
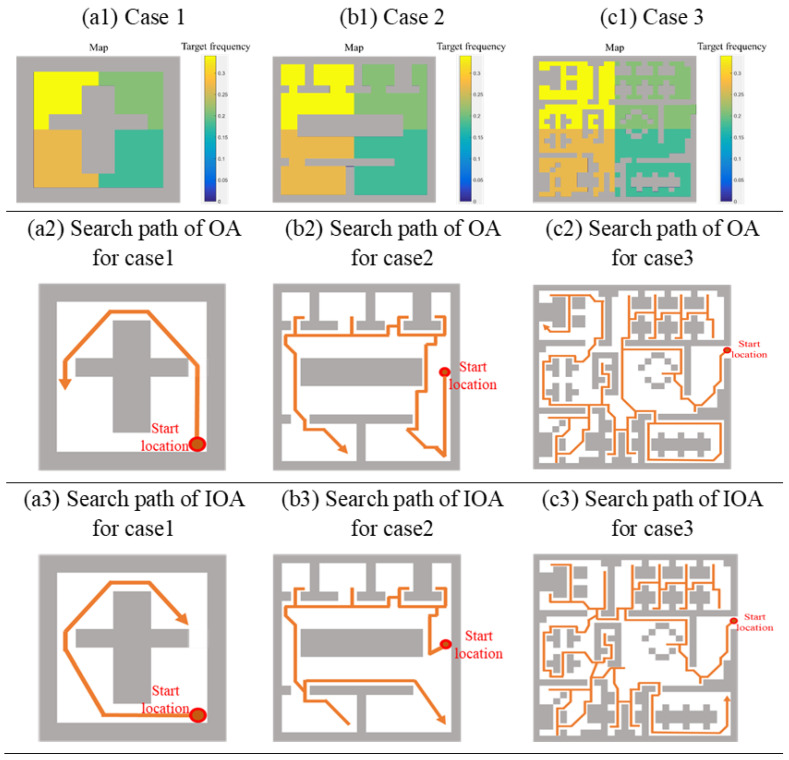
Planed paths based on an algorithm considering target frequency and not considering target frequency.

Assuming the known prior knowledge of target frequency, we use the characteristics of the Poisson distribution to simulate the frequency of the target appearing. Poisson distribution is suitable for describing the number of random events occurring per unit time [22]. We set the probability of target occurrence in the 4 sub-regions based on the Poisson distribution (*p*-value is 0.226 obtained Kolmogorov–Smirnov (K-S) test). A *p*-value (calculated by SPSS) of 0.05 or less is commonly interpreted by statistician as justification for rejecting the null hypothesis that the data are Poisson distributed. As shown in Figure 13(a1–c1), we divide the passable area of the map into four different areas according to the frequency of target appearance.

The frequency of target non-occurrence is equal to 1 minus the probability of target occurrence. The optimization algorithm for path planning of target searching considering the target frequency is defined as the improved optimization algorithm (IOA). The original OA does not take into account the frequency of the target.

As shown in Figure 13, the path that can achieve vision complete coverage of passable areas obtained by OA is shorter than the path obtained by IOA.

We exhibit the path lengths, path length increase rate of IOA to OA, average time of target searching, time reduce rate of IOA to OA of 3 cases, as shown in Table 2.

**Figure 14:**
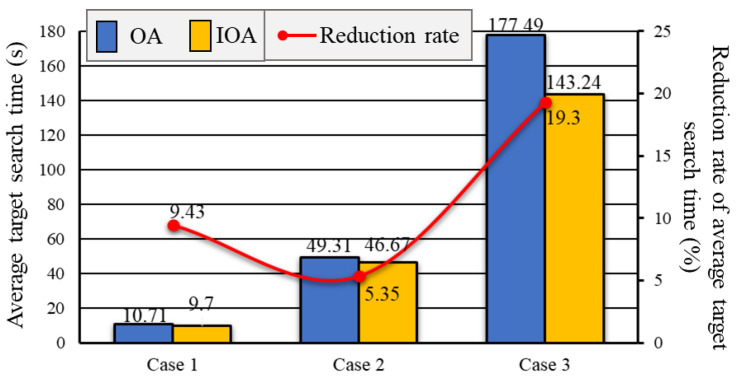
The distance reduction rate of path obtained by OA relative to RBA and GA.

The results show that IOA can effectively reduce the average time of target searching (see Figure 14). The average time of target searching in case 1 for OA is 10.71 s, and 9.7 s for IOA, which is 9.43% shorter. The average time of target searching in case 2 for OA is 49.31 s, and 46.67 s for IOA, which is 5.35% shorter. The average time of target searching in case 3 for OA is 177.49 s, and 143.24 s for IOA, which is 19.3% shorter.

### 5.4. Results of the Test in a Real Environment with a Real Robot

**Figure 15:**
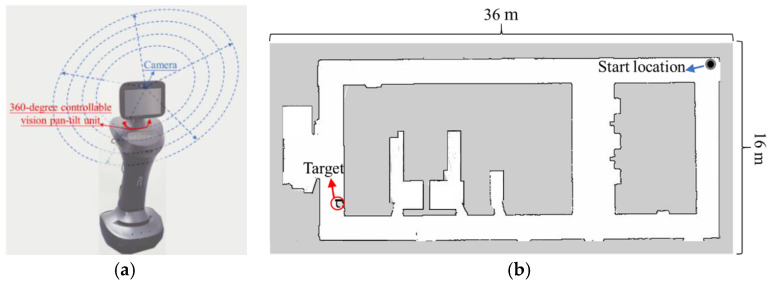
(**a**) A mobile robot for target searching; (**b**) a real test environment.

We further conduct a test in a real environment with a real robot (see Figure 15). The test environment is a corridor with many offices and some obstacles. A target exists in one place in the corridor. The test environment is about 576 square meters. The robot set off at one end of the corridor.

**Figure 16:**
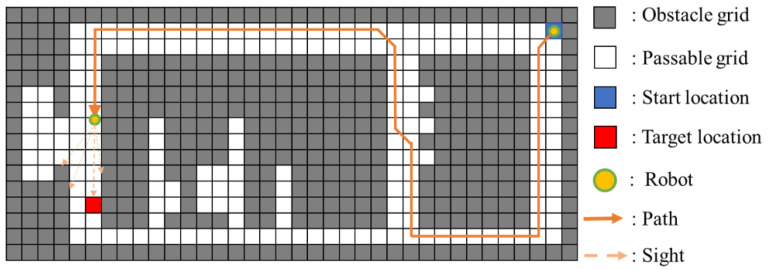
The distance reduction rate of path obtained by OA relative to RBA and GA.

The target search path is obtained through our optimization algorithm in the real test environment, as shown in Figure 16. The target search path brought by OA is 57.7 m. The time used in this target search is 115 s. This test with a real environment using a real robot further verifies the feasibility of our algorithm.

## 6. Conclusions

This paper tackles the challenging path planning problem of target search for mobile robots. We propose a two-stage optimization model to efficiently obtain an approximate optimal solution. In this model, we first develop an efficient method to determine the key locations for visual complete coverage of a two-dimensional grid map, which is constructed by drawing lessons from the method of corner detection in the image processing. As the field of view of the robot camera can completely search the entire known area, the union of the area covered by the visual field of the robot camera at the key locations can achieve full coverage of the whole environment. Then, we design a planning problem for searching the shortest path that passes all key locations considering the frequency of target occurrence. Several tests are conducted in the paper to validate the proposed sufficient method to path planning problem of target search with a mobile robot. The results show that the proposed optimization algorithm (OA) can achieve the significantly shorter search path length and the shorter target search time than the current Rule-based Algorithm (RBA) and Genetic Algorithm (GA) in various simulation cases. Furthermore, the results show that the improved optimization algorithm (IOA) with the priori known frequency of occurrence of the target can effectively search the target with shorter time compared to OA. We also set up a test in a real environment to verify the feasibility of our algorithm.

The ultimate goal of our research was to improve the mobile robots’ target searching in a complex environment with uncertainties from several elements and the dynamic obstacles. To achieve this goal, first, we needed to plan the “global” optimal path for the static environment; then, the robot decides some “local” optimal path when detecting dynamic obstacles along the global path in real time. This paper only focuses on the first part of this problem since even for this problem there are many challenging technical problems which need to be addressed. After the robot detects the dynamic obstacles in real time, the robot will plan a local path to “pass” the dynamic obstacles and then return to the global path. This part is also a challenging research topic. In the future, we will introduce a more realistic and complicated situation which considers complex dynamic environment. In addition, due to lack of multiple test data for searching the target in the working environment (no prior knowledge of target frequency), we only conducted a simple and rough treatment for the frequency of target occurrence in our testing. Verifications using more actual test data in the future could further improve our work.

## Figures and Tables

**Table 1:** Primary notation.

Parameters	
*S*	set of passable grids
*P*	optimal path
*n*	number of observation locations of *P*
*i*	the ID of the observation locations of *P*, i∈{1,⋯,n}
$p_{i}$	observation location, $p_{i}\in P$
*Z*	set of subpath of *P*
$z_{p_{i},p_{i+1}}$	a subpath which refers that robot moves from $p_{i}$ to $p_{i+1}$, $z_{p_{i},p_{i+1}}\in Z$
∪v(Z)	union of a series of visible grid sets detected by Z
$c(z_{p_{i},p_{i+1}})$	the moving cost associated with $z_{p_{i},p_{i+1}}$, $z_{p_{i},p_{i+1}}\in Z$
*g*	the ID of the starting location of a mobile robot
Ω	set of key locations
$\alpha_{k}$	a binary indicating if the grid *k* is set as a key location, k∈S
v(k)	set of passable grids that the robot can detect at key location *k*
∪v(Ω)	union of passable grids visible that the robot can detect at all key locations ∈Ω
$N_{\Omega }$	number of key locations in Ω
$x_{j,k}$	binary factor, which indicates whether the mobile robot moves from key location *j* to *k*, j,k∈Ω
d(j,k)	distance between key location *j* and *k*, j,k∈Ω
$f_{k}$	probability of the robot unable to search for the target at the key location *k*, k∈Ω
$w_{k}$	number of test times of the robot unable to search for the target at key location *k*, k∈Ω
*W*	total number of test times
Φ	subset of set Ω

**Table 2:** Path length (m), increase rate of path length (%), average time (s), and reduced rate of average time (%).

	Path Length (m)		Increase Rate of Path Length (%)	Average Time (s)		Reduce Rate of Average Time (%)
	OA	IOA		OA	IOA	
Case 1	15	18	20	10.71	9.7	9.43
Case 2	69	84	21.74	49.31	46.67	5.35
Case 3	231	234	1.3	177.49	143.24	19.3
